# A Decade of Peritoneal Dialysis Attrition: A Single-Centre Canadian Retrospective Cohort Study

**DOI:** 10.1177/20543581261478488

**Published:** 2026-09-30

**Authors:** Ashley Benchluch, Christopher Gayowsky, Bourne L. Auguste

**Affiliations:** 1 Department of Medicine, 12366 University of Toronto, Toronto, ON, Canada; 2 6221 Medical Sciences, Western University, London, ON, Canada; 3 Division of Nephrology, 71545 Sunnybrook Health Sciences Centre, Toronto, ON, Canada

**Keywords:** peritoneal dialysis, attrition, technique survival, competing risks, transplantation

## Abstract

**Background:**

Peritoneal dialysis (PD) attrition is commonly used as a marker of program quality. However, all-cause attrition encompasses competing outcomes including transplantation, death, and technique failure, which carry fundamentally different clinical and programmatic implications. Cause-specific analysis is therefore essential for accurate program evaluation.

**Objective:**

To characterize cause-specific PD attrition over a 10-year period and identify predictors of PD discontinuation using a competing risks framework.

**Design:**

Single-centre retrospective cohort study.

**Setting:**

Sunnybrook Health Sciences Centre, a tertiary academic centre in Toronto, Canada, with active referral relationships to university-affiliated kidney transplant centres.

**Patients:**

All adult patients (age ≥18 years) who initiated PD between January 1, 2014 and December 31, 2024 with a minimum of one month of follow-up (N = 230).

**Measurements:**

Cause-specific PD exit (transfer to hemodialysis [HD], kidney transplantation, death, facility transfer, kidney recovery). Among patients transferring to HD, clinical reasons for transfer were recorded. Predictors assessed included age, sex, diabetes status, PD modality, assisted PD use, and residual kidney function.

**Methods:**

Cause-specific cumulative incidence functions were estimated using the Aalen-Johansen estimator. For clarity, only the three principal causes, transfer to hemodialysis, kidney transplantation, and death, are displayed. Multivariable Cox proportional hazards models were fit within a cause-specific hazard framework to evaluate predictors of PD discontinuation.

**Results:**

During follow-up, 162 patients (70.4%) discontinued PD. In the early follow-up period, transfer to HD was the predominant cause of PD exit. Over longer follow-up, kidney transplantation emerged as the dominant cause of PD discontinuation, with its cumulative incidence exceeding that of HD transfer. Among 53 patients who transferred to HD, the most common reasons were peritonitis or other infection (37.7%), catheter non-function (20.8%), and under-dialysis (17.0%). In cause-specific analysis, male sex was independently associated with a higher hazard of technique failure (HR 3.15, 95% CI 1.62-6.12), whereas older age was associated with a lower hazard of both technique failure and transplantation but a higher hazard of death, offsetting effects that produced a null age association in a combined model. Kidney transplantation was the single most common cause of PD exit (54, 33.3%).

**Limitations:**

Single-centre retrospective design with limited sample size. Important comorbidities including cardiovascular disease and frailty were not systematically captured. Non-medical contributors to attrition were not assessed. High transplant referral activity may limit generalizability.

**Conclusions:**

In a Canadian PD program with active transplant referral relationships, kidney transplantation was the dominant driver of PD discontinuation over longer follow-up, underscoring that all-cause attrition cannot be interpreted uniformly across programs. Cause-specific, case-mix adjusted benchmarks are needed to accurately evaluate PD program performance.

## Introduction

Peritoneal dialysis (PD) is a well-established and effective modality for kidney replacement therapy and is a central component of home dialysis strategies. Compared with in-centre hemodialysis (HD), PD is associated with greater treatment flexibility and quality-of-life benefits and is promoted in many health systems as part of broader home-first initiatives.^1^ In Ontario, the Ontario Renal Network (ORN) reports home dialysis utilization as a provincial system performance indicator.^2^ The proportion of patients on chronic dialysis receiving home dialysis increased from 22.6% in 2012/2013 to 24.4% in 2023/2024, however, this remains below the provincial target of 27%.^3^ Achieving and sustaining home dialysis targets depends not only on initiating patients on PD, but also on maintaining them successfully over time. Therefore, it is important to identify potential threats to provincial home dialysis targets, including factors associated with PD discontinuation.

PD attrition remains common and multifactorial. While technique failure, commonly defined as transfer from PD to HD, is frequently examined, overall PD attrition reflects a broader set of patient and system-level factors influencing the sustainability of PD therapy. Peritonitis, infections, catheter malfunction, and ultrafiltration failure have consistently been identified as leading drivers of technique failure, while transplantation and death represent additional and competing outcomes.^4,5^ Provincial data from Ontario demonstrate wide variability in PD attrition rates across programs, with differences not fully explained by patient characteristics alone, suggesting the influence of modifiable system-level factors.^1,6^

One potential strategy to address early PD attrition is assisted peritoneal dialysis, in which trained caregivers or home-care providers support patients who face functional, cognitive, or physical barriers to self-care PD. Assisted PD has been proposed as a way to reduce technique failure among patients with reduced dexterity, frailty, or mobility limitations who may otherwise experience early failure on a self-care model.^4^ These observations raise an important care-delivery question: whether assisted PD may be associated with different attrition patterns.

This study aims to identify predictors of overall PD attrition, defined as discontinuation of PD for any cause, and to characterize cause-specific PD exits using a competing risks framework. We examined predictors including age, sex, diabetes, PD modality, assisted PD use, residual urine output, and dialysis vintage. Among those who transferred to HD, we describe the clinical reasons for modality change. By examining factors associated with PD discontinuation, this analysis seeks to inform strategies that support sustainable home dialysis care in Ontario.

## Methods

### Study Design and Setting

We conducted a single-centre retrospective cohort study at Sunnybrook Health Sciences Centre, a tertiary academic centre in Toronto, Canada, evaluating PD outcomes over a 10-year period (January 1, 2014 to December 31, 2024). Electronic medical records were reviewed using Accuro EMR. Data were abstracted through structured chart review and entered into a de-identified analytic dataset using study-specific identifiers.

### Study Population

We included all adult patients (≥18 years) who initiated PD at our centre during the study period and had a minimum of one month of follow-up data. Patients were excluded if PD use was temporary with a planned return to HD, or if insufficient baseline or outcome data precluded inclusion in the analytic dataset.

### Variables and Operational Definitions

Baseline variables collected at PD initiation included age, sex, diabetes status, PD modality (automated PD [APD] vs. continuous ambulatory PD [CAPD]), assisted PD status, PD vintage, and residual kidney function (RKF). Assisted PD was defined as receipt of community home nursing support at least once daily to assist with PD-related care tasks. RKF was ascertained using the most recent available 24-hour urine volume documented during follow-up. Patients with any recorded urine output greater than 0 mL were classified as having RKF. Patients for whom no 24-hour urine volume was documented at any point during follow-up were classified as having no RKF. This assumption may underestimate the prevalence of RKF in patients with incomplete urine collection data and represents a potential source of misclassification bias.

### Outcomes

The primary outcome was PD attrition, defined as permanent discontinuation of PD due to one of the following mutually exclusive causes: transfer to hemodialysis, death, kidney transplantation, transfer of care to another facility, or kidney recovery. Transfer to hemodialysis was classified as technique failure when it persisted beyond 30 days without return to peritoneal dialysis; transfers with return to PD within 30 days were not counted as technique failure, and death occurring within 30 days of transfer was classified as death rather than technique failure. The index date (time zero) was the date of PD initiation. Patients remaining on PD at the time of last follow-up were censored at that date.

Among patients who transferred to hemodialysis, the clinical reason for transfer was categorized based on program documentation and chart review into one of the following predefined categories: peritonitis or other infection, catheter non-function, volume overload, under-dialysis, patient preference, leak, or hernia and other surgical complications.

### Statistical Analysis

Baseline characteristics were summarized using mean (standard deviation) or median (interquartile range) for continuous variables, and counts (percentages) for categorical variables. To characterize the time-to-event distribution for each cause of PD exit while accounting for the presence of competing events, we estimated cause-specific cumulative incidence functions using the Aalen-Johansen estimator, with death, transfer to hemodialysis, kidney transplantation, facility transfer, and kidney recovery treated as competing risks. Cumulative incidence estimates were reported at prespecified time points (12, 24, and 36 months) and stratified by assisted PD status where relevant. To evaluate baseline predictors of each cause of PD exit, we fit separate cause-specific Cox proportional hazards models for transfer to hemodialysis (technique failure), kidney transplantation, and death. In each model the remaining causes of exit were treated as competing events and censored at their observed time. Covariates were selected a priori on clinical and biological grounds and constrained to approximately one covariate per ten events; PD modality was not included because 93 percent of patients received automated PD. The proportional hazards assumption was assessed for each model using scaled Schoenfeld residuals. The cause-specific hazard was chosen as the appropriate estimand for these etiologic questions, and the Fine-Gray subdistribution hazard, which targets absolute risk, was therefore not used as the primary model. Results are reported as hazard ratios (HR) with 95% confidence intervals (CI). All tests were two-sided and statistical significance was defined as p<0.05. All analyses were performed in R [version 4.4.3] (R Foundation for Statistical Computing, Vienna, Austria) using the survival and tidycmprsk [version 1.1.0] packages.

### Ethical Considerations

This study was approved by the Sunnybrook Research Ethics Board (SUN 6661). Given the retrospective design and use of de-identified data, a waiver of informed consent was granted.

## Results

### Study Sample Characteristics

A total of 230 patients initiated PD during the study period. The mean age was 63.4 years (SD 17.7), 117 (50.9%) were male, and 100 of 225 (44.4%) had diabetes. The majority of patients were initiated on automated PD (212/228, 93.0%), and 90 patients (39.1%) received assisted PD. Median residual urine output was 621 mL/24h (95% CI 533-752). Additional baseline characteristics are presented in Table 1.

**Table 1.** Baseline Characteristics of Patients Initiating Peritoneal Dialysis (N = 230)

Characteristic	Value
N	230
Age, mean (SD), years	63.4 (17.7)
Male, n (%)	117 (50.9%)
Diabetes, n/N (%)	100/225 (44.4%)
Assisted PD, n (%)	90 (39.1%)
Automated PD (APD), n/N (%)	212/228 (93.0%)
Manual PD (CAPD), n/N (%)	16/228 (7.0%)
Residual urine output, median (95% CI), mL/24h	621 (533-752)
Residual urine missing, n (%)	38 (16.5%)

### Predictors of Cause-Specific PD Exit

Cause-specific results are presented in Figure 1 and Table 2. In the model for technique failure (transfer to hemodialysis), male sex was independently associated with a substantially higher hazard (HR 3.15, 95% CI 1.62 to 6.12) and older age with a lower hazard (HR 0.80 per 10-year increment, 95% CI 0.65 to 0.98); diabetes and assisted PD were not significantly associated. In the model for kidney transplantation, older age (HR 0.71, 95% CI 0.58 to 0.88) and male sex (HR 0.51, 95% CI 0.26 to 1.00) were each associated with a lower hazard, and assisted PD showed a similar direction (HR 0.34, 95% CI 0.11 to 1.01). In the model for death, older age was strongly associated with a higher hazard (HR 2.03 per 10-year increment, 95% CI 1.53 to 2.69). The proportional hazards assumption was satisfied in all three models. The opposing effects of age across causes offset one another in a combined any-cause model, which yielded a null and uninterpretable age association (HR 0.90, 95% CI 0.80 to 1.02), illustrating why cause-specific modeling is essential to this research question.

**Figure 1.**
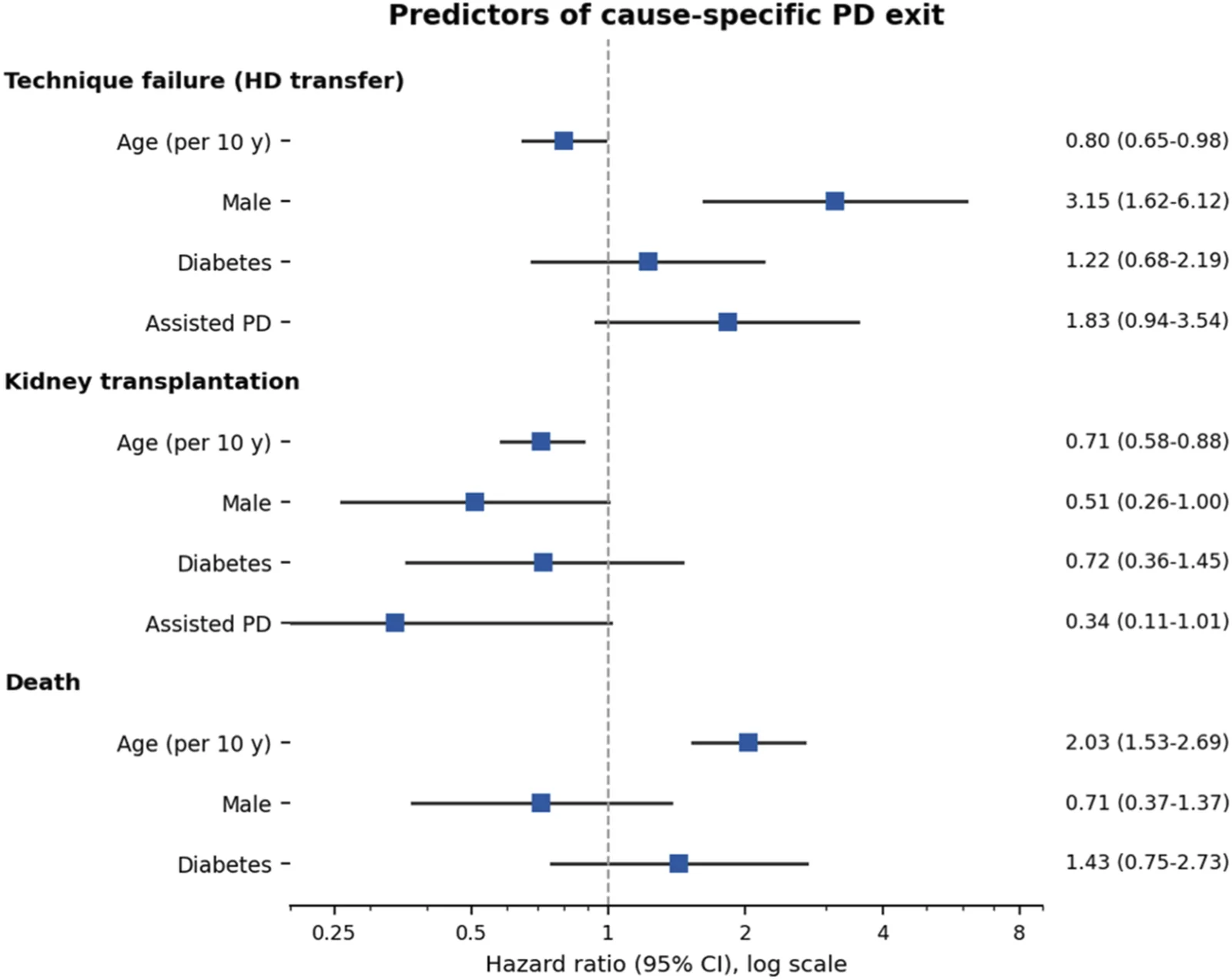
Predictors of cause-specific PD exit: results of separate cause-specific Cox proportional hazards models for technique failure (transfer to hemodialysis), kidney transplantation, and death. Results are presented as hazard ratios (HR) with 95% confidence intervals

**Table 2.** Cause-Specific Hazard Ratios for the Principal Causes of PD Exit

Predictor	HR	95% CI	p
***Technique failure (transfer to hemodialysis), 51 events***			
Age (per 10 y)	0.80	0.65-0.98	0.034
Male	3.15	1.62-6.12	0.001
Diabetes	1.22	0.68-2.19	0.503
Assisted PD	1.83	0.94-3.54	0.075
***Kidney transplantation, 54 events***			
Age (per 10 y)	0.71	0.58-0.88	0.002
Male	0.51	0.26-1.00	0.050
Diabetes	0.72	0.36-1.45	0.360
Assisted PD	0.34	0.11-1.01	0.052
***Death, 44 events***			
Age (per 10 y)	2.03	1.53-2.69	<0.001
Male	0.71	0.37-1.37	0.310
Diabetes	1.43	0.75-2.73	0.278

### Overall PD Attrition

During follow-up, 162 patients (70.4%) discontinued PD for any cause (Table 3). Early attrition was observed, with 14 patients (6.1%) discontinuing within 90 days of initiation and an additional 24 patients (10.4%) discontinuing between 91 and 180 days. The majority of attrition occurred beyond 180 days of follow-up (n=124, 53.9%). The cumulative incidence of overall PD attrition is shown in Figure 2. Attrition increased steadily following PD initiation, with the steepest rise observed during the first 24 months. By 80 months of follow-up, nearly all patients had discontinued PD.

**Table 3.** Peritoneal Dialysis Attrition and Reasons for PD Exit

Outcome	Value
**Patients discontinuing PD, n (%)**	162 (70.4%)
**Timing of discontinuation**	
0-90 days, n (%)	14 (6.1%)
91-180 days, n (%)	24 (10.4%)
>180 days, n (%)	124 (53.9%)
**Cause of PD exit**	
Transfer to hemodialysis, n (%)	53 (32.7%)
Kidney transplantation, n (%)	54 (33.3%)
Death, n (%)	44 (27.2%)
Transfer to another facility, n (%)	10 (6.2%)
Recovery of kidney function, n (%)	1 (0.6%)
**Reasons for hemodialysis transfer (among 53 patients)**	
Peritonitis or other infection	37.7%
Catheter non-function	20.8%
Under-dialysis	17.0%
Patient preference	9.4%
Leak	9.4%
Hernia or other surgical complications	5.7%
Volume overload	0.0%

**Figure 2.**
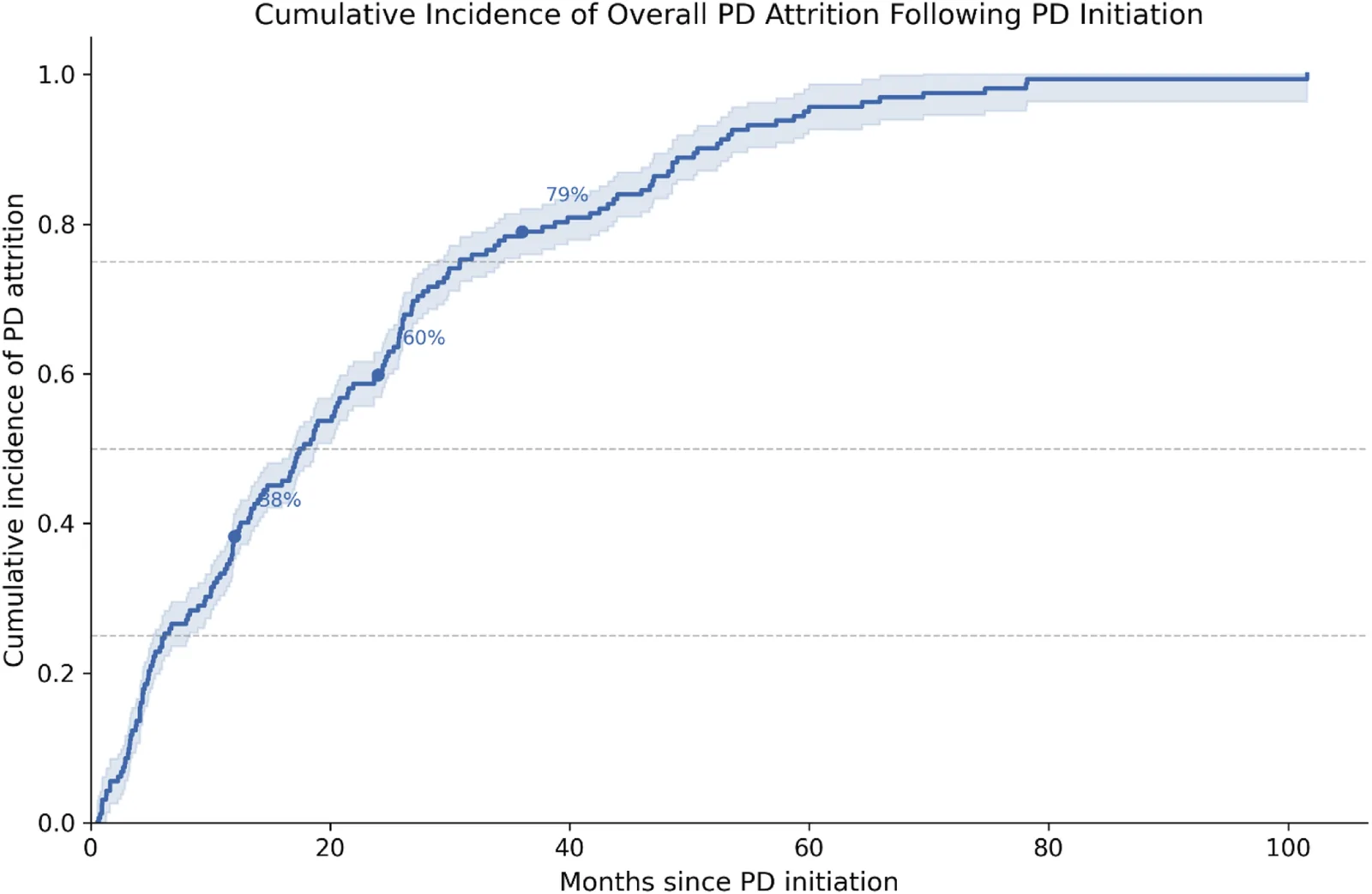
Cumulative incidence of overall peritoneal dialysis attrition following PD initiation

### Cause-Specific PD Exit

Cause-specific cumulative incidence of PD exit is presented in Figure 3. In the early follow-up period, transfer to hemodialysis was the predominant cause of PD discontinuation. However, kidney transplantation emerged as the leading cause of PD exit over longer follow-up, with its cumulative incidence exceeding that of hemodialysis transfer and remaining the dominant mode of PD discontinuation thereafter.

**Figure 3.**
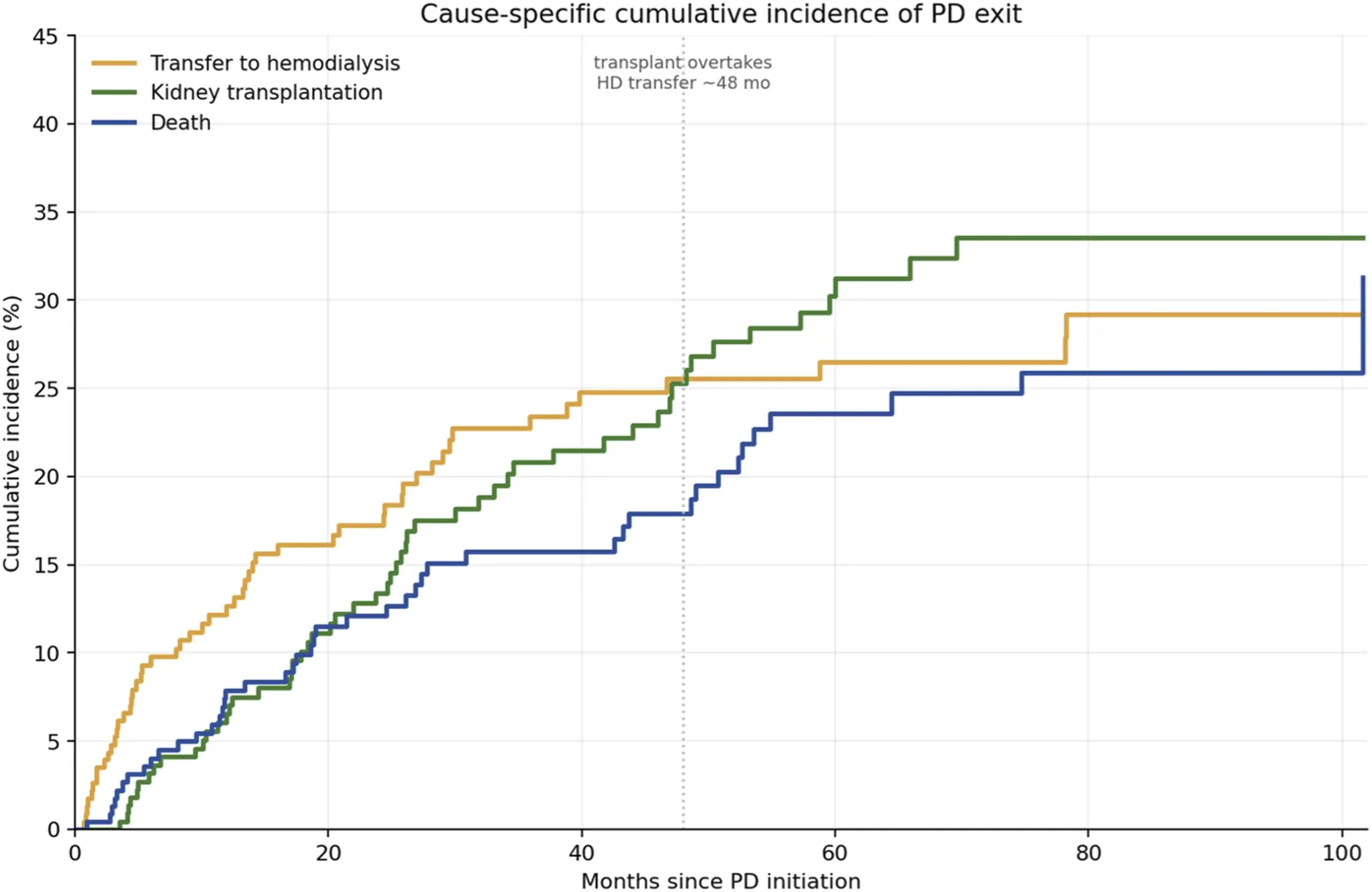
Cause-specific cumulative incidence of PD exit following PD initiation, estimated using the Aalen-Johansen estimator. Competing events include transfer to hemodialysis, kidney transplantation, death, transfer to another facility, and kidney recovery

Among the 162 patients who discontinued PD, kidney transplantation was the single most common cause (54, 33.3%), followed by transfer to hemodialysis (53, 32.7%) and death (44, 27.2%); transfer to another facility (10, 6.2%) and recovery of kidney function (1, 0.6%) accounted for the remainder (Table 3). The cumulative incidence of transplantation overtook that of hemodialysis transfer at approximately 48 months of follow-up.

### Reasons for Transfer to Hemodialysis

Among the 53 patients who transferred to hemodialysis, the most common reason was peritonitis or other infection (37.7%), followed by catheter non-function (20.8%) and under-dialysis (17.0%). Patient preference and catheter leak each accounted for 9.4% of transfers, and hernia or other surgical complications accounted for 5.7%. No transfers were attributed to volume overload.

## Discussion

PD technique survival has historically been the primary outcome used to benchmark PD program quality, with transfer to HD representing the most commonly reported adverse modality transition.^1,2^ Prior registry-based studies have identified transfer rates of approximately 10 to 20% per patient year, with infectious complications, particularly peritonitis, representing the leading modifiable driver of poor PD technique survival.^4,7^ A fundamental challenge in interpreting this literature is the lack of consensus on how discontinuation is defined: some studies include any HD transfer while others require transitions exceeding 30, 60, or 90 days, and censoring for transplantation is applied inconsistently, yielding reported discontinuation rates that range from 6 to 40% across studies.^8^ This definitional heterogeneity complicates cross-program comparisons and has been identified as a major barrier to evidence-based quality improvement in PD.^8^ Death represents a further major competing cause of PD exit, contributing approximately 25 to 30% of all discontinuation events in long-term follow-up cohorts such as NECOSAD.^5,9^

Our findings diverge from much of this literature in an important respect. While early PD discontinuation in our cohort was most commonly attributable to HD transfer, consistent with international data reporting catheter complications and infection as predominant early causes of attrition,^7,9^ transplantation emerged as the dominant cause of PD exit over longer follow up. The NECOSAD investigators observed a similar trajectory, with transplantation progressively dominating competing events over time and 30% of patients having received a kidney transplant by 12 months after PD initiation.^9^ This pattern reflects the characteristics of our centre, which, while not itself a transplant program, maintains active referral relationships with university-affiliated transplant centres in the city, resulting in a patient population with relatively high transplant candidacy. This underscores that in settings where a substantial proportion of patients are referred for and proceed to transplantation, high overall attrition may paradoxically reflect program success rather than failure, and that all-cause attrition rates cannot be interpreted uniformly across programs with differing transplant referral activity.

Among patients who transferred to HD, peritonitis and other infectious complications accounted for the largest proportion of transfers (37.7%), consistent with established literature identifying peritonitis as the leading cause of poor PD technique survival.^4,6,8^ This is supported by the ANZDATA registry, in which infections accounted for 24.0% of first-year technique failure among 16,748 patients, alongside mechanical causes (19.7%) and death (32.2%).^10^ An important refinement comes from the DOMESTICO study, which demonstrated a characteristic time-dependent pattern whereby catheter-related problems drove 15% of early discontinuation but fell to 5% after six months, while infections remained an important cause throughout, suggesting that early and late attrition reflect distinct pathophysiological processes requiring phase-specific clinical responses.^11^ Catheter non-function (20.8%) and under-dialysis (17.0%) were the next most common reasons for HD transfer in our cohort, concordant with prior reports.^5,8,9,12^ Notably, no transfers were attributed to volume overload, which may reflect patient selection, proactive ultrafiltration management, or differences in how volume-related indications are documented, and warrants cautious interpretation.

The role of assisted PD in modifying attrition patterns adds further complexity. Functional limitations, cognitive impairment, and frailty are well established barriers to self-care PD, and prospective assessment has found that up to 63% of patients without a contraindication to PD have a physical or cognitive barrier to self-care.^13^ Beyond clinical barriers, non-medical factors including caregiver loss and patient burnout are increasingly recognised as independent contributors to late discontinuation and represent potentially modifiable retention targets.^14,15^ Assisted PD has been associated with lower rates of HD transfer and, in populations with diabetes and elderly, a protective effect against peritonitis.^13,16,17^ The introduction of an assisted PD service in the United Kingdom was associated with a significantly higher rate of PD initiation (HR 1.78, 95% CI 1.21 to 2.61), suggesting that access to assistance expands the population able to receive home-based therapy.^18^ The competing outcome profile of assisted PD patients is, however, fundamentally different from that of self-care PD patients: assisted patients are typically older and frailer, with lower transplant eligibility and higher competing mortality risk, such that overall attrition becomes an unreliable performance metric for these programs without explicit case-mix adjustment.^13^ In our cohort, assisted PD was not significantly associated with technique failure, but assisted patients were markedly less likely to undergo transplantation, with a cumulative incidence of transplantation of only 1.3 percent at 36 months compared with 32.1 percent in self-care patients. This profound difference in competing-risk profile, rather than any single hazard, is what makes overall attrition an unreliable performance metric for assisted PD programs without explicit case-mix adjustment, and these subgroup findings should be interpreted cautiously given limited power.

Smaller centre size and late nephrology referral have been consistently identified as independent risk factors for early PD discontinuation across the French, ANZDATA, and New Zealand registry datasets, likely reflecting differences in staff experience, training capacity, and access to quality improvement infrastructure.^8,10,19^ These volume-outcome relationships have important implications for how regional PD services are organised and funded. Age-related differences in PD technique survival have been reported inconsistently in the literature, with some cohorts demonstrating similar one-year HD transfer rates across age groups.^6^ While older age has been associated with higher discontinuation risk in multiple large registries, this association likely reflects greater comorbidity burden and competing mortality risk rather than a direct effect of age on PD sustainability.^5,8,14^ In our cohort, older age carried opposing cause-specific effects: it lowered the hazard of both technique failure (HR 0.80 per 10 years) and transplantation (HR 0.71), the latter reflecting the lower transplant candidacy of older patients, while it sharply raised the hazard of death (HR 2.03 per 10 years). These offsetting effects explain why age appeared unrelated to attrition in a combined model and can only be disentangled with cause-specific analysis. This observation reinforces the importance of cause-specific competing risks analysis when interpreting age-related predictors of PD attrition, particularly in programs with active transplant referral relationships.

Male sex was independently associated with a significantly higher cause-specific hazard of technique failure in our analysis (HR 3.15, 95% CI 1.62 to 6.12), and was not associated with a higher hazard of transplantation or death, consistent with prior literature citing higher cardiovascular comorbidity, diabetes-related complications, and smoking-related disease as proposed mechanisms.^20^ In a large Singaporean multiethnic cohort, male sex was specifically associated with higher rates of PD-related infections, contributing to approximately 7.7% of all discontinuation events,^21^ and the DOMESTICO study identified ischemic heart disease as an independent predictor of technique failure.^11^ Cardiovascular comorbidity and smoking status were not included as covariates in our model, and residual confounding by these factors cannot be excluded. The wide confidence interval reflects the sample size limitations of this study, and this finding warrants replication in larger cohorts.

Taken together, these findings highlight a fundamental limitation of conventional PD program evaluation. Collapsing transplantation, death, and HD transfer into a single attrition metric conflates program quality with patient case mix and obscures the competing event structure that underlies outcomes in any given program. Programs with active transplant referral relationships serving younger, more transplant-eligible patients will generate high apparent attrition not through poor technique survival, but through successful progression to transplantation. The development of cause-specific, case-mix adjusted benchmarks for PD program performance represents an important and as yet unmet priority for the field.^8,13^

## Limitations

Several limitations of this study warrant acknowledgment. This was a single centre retrospective analysis with a relatively small sample size of 230 patients, which limits statistical power and may have precluded detection of associations that would be apparent in larger cohorts. This is particularly relevant for subgroup analyses such as assisted PD, where the competing outcome structure further reduces effective sample size, a challenge noted even in substantially larger registry-based studies.^8,10^ The wide confidence intervals observed for several predictors, including the male sex hazard ratio, reflect this limitation. In addition, residual kidney function was ascertained from available 24-hour urine collections, and patients without any documented urine volume were assumed to have no residual function. This assumption may have introduced misclassification bias, potentially underestimating the prevalence of residual kidney function, a variable with known prognostic importance for technique survival particularly in the second year of PD.^9^ Prior exposure to hemodialysis, a recognized predictor of PD technique failure that correlates closely with residual kidney function, was not available in our dataset and could not be included as a covariate, representing a further potential source of residual confounding in the technique-failure model. Moreover, important comorbidities including cardiovascular disease, frailty, and functional status were not systematically captured as covariates. Given that comorbidity burden, ischemic heart disease, and frailty have been identified as independent predictors of PD discontinuation across multiple large cohorts, their absence introduces the possibility of residual confounding.^10,11^ Fourth, non-medical factors contributing to PD discontinuation including caregiver availability, patient burnout, and loss of confidence in PD were not captured, despite evidence that these represent meaningful and potentially modifiable drivers of late attrition.^14,15^ Fifth, the proportion of patients referred to and proceeding to transplantation reflects the characteristics of our referral relationships with affiliated university transplant centres and the case mix of our patient population, and may not be generalizable to programs with different referral patterns or transplant access. Finally, the retrospective design introduces the possibility of documentation bias in the ascertainment of reasons for HD transfer, a limitation inherent to most single-centre PD outcome studies and one that argues for prospective, standardised cause-of-exit coding in future work.^8^ Despite these limitations, this study provides 10-year cause-specific attrition data from a Canadian PD program using competing risks methodology, contributing to a growing body of evidence supporting the need for more nuanced approaches to PD program evaluation.

## Conclusions

Quality evaluation of PD programs must move beyond overall attrition rates and incorporate cause-specific competing risks analyses that distinguish poor PD technique survival from expected and desirable modality transitions. In our cohort, kidney transplantation emerged as the leading driver of PD discontinuation over longer follow up, a finding attributable in part to our centre’s active referral relationships with university-affiliated transplant centres and the relatively high transplant candidacy of our patient population. Interpreting all PD discontinuation as evidence of poor technique survival risks misclassifying successful progression along the kidney replacement pathway as program failure, with real consequences for how programs are evaluated and resourced. Provincial and national quality metrics that rely on raw attrition rates may systematically disadvantage programs serving populations with higher transplant candidacy. Future multicentre studies using competing risks methodology will be essential to develop accurate, case-mix adjusted benchmarks for PD program performance and to distinguish modifiable determinants of technique survival from appropriate transitions across the kidney replacement pathway.

## Supplemental Material

Supplemental Material - A Decade of Peritoneal Dialysis Attrition: A Single-Centre Canadian Retrospective Cohort Study Supplemental Material for A Decade of Peritoneal Dialysis Attrition: A Single-Centre Canadian Retrospective Cohort Study by Ashley Benchluch, Christopher Gayowsky, Bourne L. Auguste, MD, MSc in Canadian Journal of Kidney Health and Disease.

## Data Availability

The data underlying this study contain potentially identifying patient information and are therefore not publicly available. Data may be made available from the corresponding author upon reasonable request, subject to institutional and ethics approval.*
