# Supplementary material for: A Decade of Peritoneal Dialysis Attrition: A Single-Centre Canadian Retrospective Cohort Study
Source: Can J Kidney Health Dis. 2026 Sep 30;13:20543581261478488. doi: 10.1177/20543581261478488 (PMC13769995; doi:10.1177/20543581261478488)
Supplement: Supplementary file 1 [file sj-pdf-1-cjk-10.1177_20543581261478488.pdf]

CJKHD-26-0052.R1

A Decade of Peritoneal Dialysis Attrition: A Single-Centre Canadian Retrospective Cohort Study

Résumé

Contexte : L'abandon de la dialyse péritonéale (DP) est un critère couramment utilisé comme indicateur de la qualité des programmes. L'abandon toutes causes confondues englobe des événements concurrents, notamment la transplantation, le décès et l'échec de la modalité, qui ont des implications cliniques et organisationnelles distinctes. Une analyse spécifique à la cause est donc essentielle pour une meilleure évaluation des programmes.

Objectif : Décrire l'abandon de la DP selon la cause sur une période de dix ans et en déterminer les facteurs prédictifs à l'aide d'un modèle de risques concurrents.

Conception : Étude de cohorte rétrospective menée dans un seul centre.

Cadre : Le Sunnybrook Health Sciences Centre, un centre universitaire de soins tertiaires à Toronto (Canada) qui entretient des liens d'aiguillage actifs avec des centres de transplantation rénale affiliés à des universités.

Sujets : Tous les patients adultes (≥ 18 ans) qui ont amorcé la DP entre le 1er janvier 2014 et le 31 décembre 2024, avec au moins un mois de suivi (n = 230).

Mesures : L'arrêt de la DP selon la cause (transfert en hémodialyse [HD], transplantation rénale, décès, transfert en établissement, récupération de la fonction rénale). Pour les patients passés à l'HD, les raisons cliniques ayant justifié le transfert ont été enregistrées. Les facteurs prédictifs évalués comprenaient l'âge, le sexe, le statut diabétique, la modalité de DP, le recours à la DP assistée et la fonction rénale résiduelle.

Méthodologie : Les fonctions d'incidence cumulée par cause ont été estimées à l'aide de l'estimateur d'Aalen-Johansen. Par souci de clarté, seules les trois principales causes (transfert vers l'HD, transplantation rénale et décès) sont présentées. Des modèles multivariables de Cox spécifiques à la cause ont été utilisés pour évaluer les facteurs prédictifs de l'arrêt de la DP.

Résultats : Au cours de la période de suivi, 162 patients (70 %) ont cessé la DP. Initialement, le transfert vers l'HD en était la principale cause. Au cours du suivi prolongé, la transplantation rénale est devenue la cause prédominante, son incidence cumulée dépassant celle du transfert vers l'HD. Parmi les 53 patients transférés en HD, la péritonite ou une autre infection (37 %), le dysfonctionnement du cathéter (20,8 %) ou une dialyse inadéquate (17,0 %) en étaient les principales raisons. Dans l'analyse par cause, le sexe masculin était indépendamment associé à un risque accru d'échec de la modalité (RR : 3,15; IC 95 % : 1,62–6,12). Un âge plus avancé était associé à un risque plus faible d'échec de la modalité et de transplantation rénale, mais à un risque plus élevé de décès, ce qui s'est traduit par l'absence d'association entre l'âge et l'arrêt global de la DP dans le modèle combiné. La transplantation rénale était la cause la plus fréquente d'arrêt de la DP (n=54; 33 %).

Limites : Étude rétrospective monocentrique avec échantillon de taille limitée. Certaines comorbidités importantes, notamment les maladies cardiovasculaires et la fragilité, n'ont pas été systématiquement documentées. Les facteurs non médicaux contribuant à l'abandon de la DP n'ont pas été évalués. Le volume élevé d'aiguillage en transplantation pourrait limiter la généralisabilité.

Conclusion : Dans un programme canadien de dialyse péritonéale (DP) entretenant des liens actifs d'aiguillage pour la transplantation rénale, celle-ci est devenue la principale cause d'arrêt de la DP au cours d'un suivi prolongé. Ces résultats montrent que l'abandon toutes causes confondues ne devrait pas être interprété de façon uniforme entre les programmes. L'évaluation de la performance des programmes de DP nécessite des critères de référence fondés sur les causes et ajustés en fonction de la composition des cas.

Enregistrement de l'essai : Non enregistré.
